# Unfamiliar personal protective equipment: The role of routine practice and other factors affecting healthcare personnel doffing strategies

**DOI:** 10.1017/ice.2023.50

**Published:** 2023-12

**Authors:** Emily E. Chasco, Jaqueline Pereira da Silva, Kimberly Dukes, Jure Baloh, Melissa Ward, Hugh P. Salehi, Heather Schacht Reisinger, Priyadarshini R. Pennathur, Loreen Herwaldt

**Affiliations:** 1 Institute for Clinical and Translational Science, University of Iowa, Iowa City, Iowa; 2 Center for Access and Delivery Research and Evaluation (CADRE), Iowa City VA Health Care System, Iowa City, Iowa; 3 Department of Internal Medicine, Carver College of Medicine, University of Iowa, Iowa City, Iowa; 4 Department of Industrial and Systems Engineering, College of Engineering, University of Iowa, Iowa City, Iowa; 5 Department of Community and Behavioral Health, College of Public Health, University of Iowa, Iowa City, Iowa; 6 Department of Health Policy and Management, University of Arkansas for Medical Sciences, Little Rock, Arkansas; 7 Department of Engineering Education, The Ohio State University, Columbus, Ohio; 8 Department of Industrial, Manufacturing and Systems Engineering, University of Texas at El Paso, El Paso, Texas; 9 Department of Epidemiology, College of Public Health, University of Iowa, Iowa City, Iowa

## Abstract

**Background:**

Healthcare personnel (HCP) may encounter unfamiliar personal protective equipment (PPE) during clinical duties, yet we know little about their doffing strategies in such situations.

**Objective:**

To better understand how HCP navigate encounters with unfamiliar PPE and the factors that influence their doffing strategies.

**Setting:**

The study was conducted at 2 Midwestern academic hospitals.

**Participants:**

The study included 70 HCP: 24 physicians and resident physicians, 31 nurses, 5 medical or nursing students, and 10 other staff. Among them, 20 had special isolation unit training.

**Methods:**

Participants completed 1 of 4 doffing simulation scenarios involving 3 mask designs, 2 gown designs, 2 glove designs, and a full PPE ensemble. Doffing simulations were video-recorded and reviewed with participants during think-aloud interviews. Interviews were audio-recorded and analyzed using thematic analysis.

**Results:**

Participants identified familiarity with PPE items and designs as an important factor in doffing. When encountering unfamiliar PPE, participants cited aspects of their routine practices such as designs typically used, donning and doffing frequency, and design cues, and their training as impacting their doffing strategies. Furthermore, they identified nonintuitive design and lack of training as barriers to doffing unfamiliar PPE appropriately.

**Conclusion:**

PPE designs may not be interchangeable, and their use may not be intuitive. HCP drew on routine practices, experiences with familiar PPE, and training to adapt doffing strategies for unfamiliar PPE. In doing so, HCP sometimes deviated from best practices meant to prevent self-contamination. Hospital policies and procedures should include ongoing and/or just-in-time training to ensure HCP are equipped to doff different PPE designs encountered during clinical care.

Proper personal protective equipment (PPE) doffing prevents pathogen spread and healthcare personnel (HCP) self-contamination.^1^ Previous research has documented that HCP self-contamination is common, with rates ranging from 46% to 90% in empirical studies.^2–4^ Furthermore, HCP regularly make critical doffing errors and contaminate themselves and the environment even when they think they doffed proficiently.^2,5–8^

Complicating matters, HCP may encounter an array of PPE items (eg, gloves, gowns, masks) and designs (eg, gowns with breakaway neck closures vs tape-tab closures), particularly if they work across clinics or facilities. PPE shortages, such as those experienced during the COVID-19 pandemic, may also affect the supply and designs available.^7^ Despite the variety of PPE designs HCP may encounter, we know little about how HCP think about and strategize doffing unfamiliar PPE.

We investigated the following: (1) how different designs of the same PPE item affect the risk of HCP self-contamination while doffing (simulations 1–3) and (2) how HCP training and experience affect their ability to doff without self-contamination (simulation 4). This research included simulated doffing scenarios followed by think-aloud interviews during which HCP described their doffing strategies and thought processes. We previously published findings from simulations 1–3 regarding factors that influence HCP doffing strategies and general barriers and facilitators to proper doffing.^9^ However, these initial analyses led us to further explore an important issue that emerged from the data, namely, how HCP perceive and think through the process of doffing unfamiliar PPE. In this manuscript, we share qualitative findings related to how HCP navigated encounters with unfamiliar PPE during simulations and the influence of routine practices, experience with familiar PPE, and training on their doffing strategies.

## Methods

### Study design and setting

As part of a large mixed-methods study, we conducted simulated PPE doffing scenarios (ie, simulations 1–4) and think-aloud interviews in 2 Midwestern academic hospitals (hospitals A and B) from September 2017 to May 2019. During simulations 1–3, we observed HCP doffing different designs of the same PPE item (ie, 3 mask designs, 2 gown designs, and 2 glove designs).^9^ During simulation 4, we observed HCP doffing identical full PPE ensembles (ie, mask, gown, and gloves) under 2 different conditions. Table 1 summarizes the simulation scenarios and the PPE used in each. We conducted simulations in clinical education and training facilities at each hospital. The Institutional Review Board at the University of Iowa approved all study activities and participants provided consent before participation.

**Table 1.** Doffing Simulation Descriptions and PPE Used

Simulation	Description	PPE Used
(1) Mask + gloves	Participants donned and doffed 3 different mask designs, and a pair of “standard” exam gloves.	Procedure mask with ear loops
		Surgical mask with ties
		Pouch-style mask with elastic headband
		+ Nonsterile nitrile exam gloves
(2) Gown + gloves	Participants donned and doffed 2 different gown designs, and a pair of “standard” exam gloves.	Over-the-head isolation gown with breakaway neck closure and thumb loops
		Isolation gown with tape-tab neck closure and elastic cuffs
		+ Nonsterile nitrile exam gloves
(3) Gloves only	Participants donned and doffed 2 different glove designs.	Nonsterile nitrile exam gloves
		Doffy gloves
(4) Full ensemble (mask, gown, gloves)	Participants donned and doffed a full PPE ensemble that included identical gown, gloves, and mask designs, under 2 different conditions (distraction vs nondistraction).^a^	Surgical mask with ties
		Over-the-head isolation gown with breakaway neck closure and thumb loops
		Nonsterile nitrile exam gloves

Note: PPE, personal protective equipment.

a The “distraction” consisted of a team member casually asking the participant questions (eg, “How has your day been?”) while they doffed.

### Sample and data collection

We recruited 70 HCP to participate in simulations through emails sent to staff and information disseminated during staff meetings (hospitals A and B) and through posters placed in rooms where physicians do their documentation (hospital A). Participants in simulations 1–3 (n = 30; 10 per simulation) were recruited from hospital A and included HCP (eg, nurses, physicians, respiratory therapists) and medical and nursing students on clinical rotations. We designed simulation 4 (n = 40) to evaluate the effects of training and experience on doffing. We recruited 20 HCP and medical and nursing students who did not have special training as well as 10 HCP who had completed special isolation unit (SIU) training at hospital A and 10 HCP from the biocontainment unit at hospital B. We excluded HCP who did not use PPE at work and students not on clinical rotation. We assigned participants to 1 of 4 simulations and collected demographic information.

### Simulations

We previously described simulation 1–3 procedures.^9^ Notably, simulation 3 participants donned and doffed standard exam gloves and Doffy gloves, which have a tab at the wrist. Because the Doffy design was novel to all participants, team members shared that the tab was designed as a doffing aid. Participants were then asked to doff once, to watch a brief video that demonstrated proper Doffy glove doffing technique, and to doff a second time. This was the only PPE item for which the study team provided guidance.

Simulation 4 procedures were like simulations 1–3 with these exceptions: (1) participants donned and doffed identical PPE ensembles (ie, mask, gown, and gloves) twice, rather than different designs of the same item and (2) participants donned and doffed under 2 conditions (distraction vs nondistraction with the condition order assigned randomly). For the “distraction,” a team member casually asked participants questions (eg, “How has your day been?”) during doffing. Methods used to assess (ie, blacklight) and document (ie, digital camera) baseline fluorescence and self-contamination with Glo Germ fluorescent marker following each episode remained unchanged. As in simulations 1–3, we video-recorded the simulation from 4 angles, and for this report, we used interview data from all 4 simulations.

### Interviews

Immediately following simulations, each participant completed an audio-recorded think-aloud interview^10–13^ during which they watched their recorded doffing episodes and described and reflected on their performances. Interviewers provided brief instructions and probed to encourage participants to expand or clarify responses (Table 2). Qualitative researchers (E.C., K.D., and J.B.) acted as the primary interviewers, but other team members with clinical patient care, infectious disease, or human factors engineering expertise (L.H., J.P., and H.S.) also asked questions.

**Table 2.** Think-Aloud Interview Script Questions and Probes

Explain the think-aloud to participants. Script/key points:
• We will now watch a video of your doffing. In as much detail as possible, talk us through it as we watch:
○ Explain the process to us as if we were complete doffing novices or lay people.
○ Describe what you were thinking and paying attention to at each step of the process.
○ This will help us understand critical moments and identify common problems in doffing.
• If you’d like to stop or rewind the video, just say “Stop.” I may also stop and ask follow-up questions.
**Let them talk and, as necessary, ask probing and follow-up questions:**
• Could you describe what you were thinking when you performed this task?
• What were you paying attention to here?
• What was the obstacle/what issues did you have here?
• What made this part easy/hard for you?
• Where, when, and why do you think you self-contaminated?
*• You may have noticed that we asked you a question during your [first or second] doffing.^a^*
* ○ Do you think that affected your performance doffing? How?*
* ○ Did that interrupt your workflow?*
* ○ Did it interrupt your thinking?*
* ○ Did it increase your mental workload?*

a Italicized questions only applied to simulation 4 (ie, distraction or nondistraction). Outcomes related to distraction vs nondistraction are not addressed in this paper.

### Data analysis

We transcribed and uploaded interviews to MAXQDA qualitative data management software.^14^ Baloh et al^9^ previously described our codebook development and analyses for simulations 1–3 (first 30 participants). We inductively developed the codes *familiarity* and *training* to capture emergent themes related to factors that influence HCP doffing strategies. Given that simulation 4 (final 40 participants) differed from previous simulations in important ways (ie, doffing identical ensembles under 2 conditions), we anticipated that codebook refinement might be necessary.

For simulation 4 data, 2 coders (E.C. and J.P.) applied the existing codebook to 2 transcripts and used open coding and memos to document data that did not fit within existing definitions. After comparing their coding, they added and defined several codes, including *routine practice*, and then applied the updated codebook to 2 additional transcripts to test fit. One coder (E.C.) coded all simulation 4 transcripts using the final codebook and examined data from simulations 1–3 to identify additional data that met the *routine practice* code definition. To increase reliability, the second coder (J.P). coded 8 (20.0%) of 40 simulation 4 transcripts and met periodically with the first coder to compare coding. They consistently had high agreement, discussed discrepancies to reach consensus, and documented connections between emergent themes related to doffing unfamiliar PPE in analytical memos.

## Results

Table 3 reports participants’ characteristics. When analyzing interview data, we inductively identified 3 interconnected themes, which provide insight into how HCP navigate doffing unfamiliar PPE: (1) (lack of) familiarity with PPE items or designs, (2) influence of routine practice, and (3) training experiences and needs. We describe these themes below and share supporting quotations in Table 4.

**Table 3.** Study Participants’ Characteristics by Simulation Scenario

Characteristic	By Simulation Scenario, No. (%)				
	Mask and Gloves	Gown and Gloves	Gloves Only	Full Ensemble	Total
	(*n*=10)	(*n*=10)	(*n*=10)	(*n*=40)	(*n*=70)
Age, average y (range)	42.7 (20–62)	28.9 (21–52)	36.4 (25–66)	40.7 (23–61)^a^	38.6 (20–66)
**Sex, n (%)**					
Female	7 (70.0)	9 (90.0)	8 (80.0)	21 (52.5)	45 (64.3)
Male	3 (30.0)	1 (10.0)	2 (20.0)	19 (47.5)	25 (35.7)
**Healthcare personnel type, n (%)**					
Nurse	3 (30.0)	6 (60.0)	6 (60.0)	16 (40.0)	31 (44.3)
Physician/Resident	3 (30.0)	1 (10.0)	2 (20.0)	18 (45.0)	24 (34.3)
Student	1 (10.0)	3 (30.0)	1 (10.0)	0 (0.0)	5 (7.1)
Other^b^	3 (30.0)	0 (0.0)	1 (10.0)	6 (15.0)	10 (14.3)
Years of experience, n (%)					
<1 y	0 (0.0)	2 (20.0)	1 (10.0)	1 (2.5)	4 (5.7)
1–5 y	2 (20.0)	5 (50.0)	3 (30.0)	11 (27.5)	21 (30.0)
5–10 y	3 (30.0)	1 (10.0)	3 (30.0)	4 (10.0)	11 (15.7)
10–15 y	0 (0.0)	1 (10.0)	1 (10.0)	7 (17.5)	9 (12.9)
>15 y	5 (50.0)	1 (10.0)	2 (20.0)	17 (42.5)	25 (35.7)
Prior training, n (%)					
PPE donning PPE doffing	9 (90.0)	8 (80.0)	9 (90.0)	37 (92.5) 35 (87.5)^c^	63 (90.0) 61 (87.1)
Hand hygiene	10 (100.0)	10 (100.0)	10 (100.0)	40 (100.0)	70 (100.0)
Special isolation unit	0 (0.0)	0 (0.0)	0 (0.0)	20 (50.0)^d^	20 (28.6)

Note: PPE, personal protective equipment.

a Mean and range reported for 39 participants for simulation 4 and 69 participants for total; 1 participant did not report age.

b Other, eg, nursing assistant, physician assistant, respiratory therapist, clinical pharmacist, nurse practitioner.

c Prior training on PPE donning reported separately from doffing for simulation 4 and total; 2 simulation 4 participants provided different responses for donning versus doffing training.

d Hospital A (n=10); hospital B (n=10).

**Table 4.** Think-Aloud Interview Themes and Subthemes with Illustrative Quotations^a^

Healthcare Personnel	Illustrative Quotation
**Theme 1. (Lack of) Familiarity with PPE items or designs**	
P03, nurse, hospital A	I’ve never used [surgical masks] in practice… I’ve seen them like, on movies. Movies, I guess surgical movies.
P63, other HCP, hospital B	(laughs) I’m more used to the gowns that tie at the neck… I’m not familiar with those [breakaway neck] gowns.
P15, physician, hospital A	Well, I don’t use this, [it’s] not the type of gown that we have in the hospital. I mean different institutes may have different gowns.
P68, physician, hospital B	We’ve used different models in different places or, you know, I see patients in different hospitals and so I, I have seen that one before, you know.
P48, physician, hospital A	… I guess I use whatever’s in the cart, but I think most the times it’s the yellow [gown]. I mean there are those blue ones that float around every once in a while but they’re kind of the same.
P13, nurse, hospital A	I’ve worked at a few different hospitals and everyone has something a little bit different… ’cause I’ve been working here for like 2 and a half years, so it’s just I’m like more used to the materials that they have here at this point.
P51, nurse, hospital A	So we had to do a tie mask, kind of like this one, but it didn’t have the sticky part. It just was a little bit different. But, yeah, I was a little frustrated and I felt like it took more time to figure out how do it right. And I’m not totally confident that I took it off the right way every time.
P19, nurse, hospital A	So usually… I pull up here and I get that off, but I think I was confused… because this is a different gown, so I took off my gloves first and then I went this way… I did it backwards of what I normally would do.
P70, other HCP, hospital B	No, we have the thumb things on some of our gowns but we don’t have that neck thing… I mean, do you normally, you tear it, or what do you do with that?
P63, other HCP, hospital B	Ours don’t have the thumb things so that kind of threw me off a little bit… I wanted to take my gloves off first ‘cause that’s gonna be the dirtiest thing when I come out of a room, but then I was like, well if it’s got the thing over my thumb, like… am I going to contaminate my wrists when I try to, when I was doing this part here, where I was trying to lift my um hands up…
P33, physician, hospital A	‘Cause I’m not used to taking ‘em off, so I was trying to remember like which order I’m supposed to take ‘em off and where to touch and where not to, not supposed to touch.
P62, nurse, hospital B	… I’m trying to think through this… again, this new process, this new outfit, what’s the best way I can do this, trying to touch the least amount of times the outer part of the gown, which clearly is the contaminated part of the gown.
**Theme 2. Influence of routine practice**	
P29, nurse, hospital A	So I think for people, depending on what their practices are, it would be a big change. I think gloves people put on and off so often that the change how you do it would uh--, it’s a lot of muscle memory to change.
P53, physician, hospital A	Well so part of it was (laughs) since we’ve been doing all of the Ebola training recently, we remove those gowns differently. And we have a very specific way that we do that that’s different than these gowns. So, typically, with these gowns we just sort of grab and just pull it off and it breaks.
P61, physician, hospital B	Yeah, so that’s… something that we teach in the biocontainment unit. It’s something that I’ve done, you know, so I worked in containment laboratories before I worked clinically in containment, and that’s always been something that we’ve done, just to limit the amount of spray and splatter.
P18, nurse, hospital A	It’s very similar to gowns I’ve used before, where they have like the Velcro, one on each side, where they Velcro together, so I knew that it had to connect somehow, and that side was the only place that had, you know, something on it, so I knew I had to get sticky somehow.
P63, other HCP, hospital B	I’m more used to the gowns that tie at the neck, and so I was trying to look at these and see if … there was a tab, but then it didn’t seem like it was sticky, so then I was like well maybe it’s not a tab, I don’t know if I’m supposed to tear this…
P23, physician, hospital A	But I kept thinking, “How am I going to pull the next [Doffy] tab, on this side?” ‘Cause I always took off one glove and then the other, so I kept thinking, ‘Oh, how, well I can’t touch this tab, so what will I do?”
**Theme 3. Training experience and needs**	
P30, physician, hospital A	… I’m sure I’ve been trained at some point… I mean I developed this, the way I do it somehow, I’m sure.
P07, other HCP, hospital A	The one I use most within the hospital is the yellow one, so for this one and the last one especially I kind of had to think about it from when I learned how to put it on… First time using it but I was uh, taught how to use it.
P22, nurse, hospital A	I don’t know, I guess in college we learned and then, kind of like a habit, so I would say…
P20, student, hospital A	… We were trained to do it like one or two ways, to take the gloves off while you’re putting the gown in the trash, or take the gown off and then take the gloves off. So, I prefer to take the gown off and then the gloves, ‘cause I feel like I’m still touching things when I’m taking the gloves off and the gown.
P32, nurse, hospital A	… Well, we had it in school and then during orientation I think they briefly went over it but we never did a hands-on put-on put-off.
P51, nurse, hospital A	I think maybe the box had a brief little picture on it. I can’t remember so well how it all went. I don’t think anyone showed me. They just said you have to tie it, so I figured it out. I was happy when we went back to the other masks.
P23, physician, hospital A	…I only watched the video once, but… if I use it more, I think I would be more comfortable with the little tab that I pull, and… pulling this off if, you know, I’ve done it a few more times… with practice I suppose.
P30, physician, hospital A	Oh, I just think it would be practice more than anything. Just kind of getting used to how to get the glove into that beak position um… I don’t know that there’d be any verbal cues or instructions that would do that.
P34, other HCP, HOSPITAL A	Um maybe, there’s probably a video but nothing like a live—where you actually practice. Which is obviously more useful for learning, muscle memory.

Note: HCP, healthcare personnel; N-95, N-95 respirator.

a Quotations have been lightly edited to remove word repetitions (eg, stammering) and verbal hesitations (eg, “um”).

### Theme 1. (Lack of) Familiarity with PPE items or designs

All participants used PPE in their work, but the specific items and designs differed by role, unit, and hospital. Many described encountering a PPE design during the simulation that was unfamiliar or that they used infrequently. For example, most participants reported that they typically used procedure masks with ear loops rather than surgical masks with ties. Hospital A participants typically used disposable gowns with breakaway neck closures and thumb loops, whereas those at hospital B wore reusable cloth gowns with neck ties. Some participants worked in settings that rarely required them to use PPE other than nitrile exam gloves. Multiple participants shared that using unfamiliar PPE was a doffing barrier during simulations.

Specific designs were truly novel for some participants (eg, Doffy gloves,^15^ pouch-style masks with elastic headbands). More often, participants had used certain designs during their education and training but not during their recent practice. Some stated that they used different PPE designs when they worked in other facilities or when hospitals obtained new designs due to shortages or changes in purchasing agreements. Participants reported encountering the greatest variation in mask and gown designs.

Although some participants believed that they retained the ability to doff infrequently used PPE properly, most who addressed this issue said they were less comfortable with designs they used infrequently. These participants reported that they struggled to draw on muscle memory or to remember their early training when doffing these items during the simulations. A minority shared that they would seek out familiar or preferred PPE designs in other areas if these were not stocked where they worked.

Participants also noted that proper doffing procedures for unfamiliar PPE were not always intuitive. For example, participants at hospital B were uncertain which strategies to use when donning and doffing the gown with a perforated neck closure, thumb loops, and side-tie belt. Those who did not use surgical masks regularly had a similar response to this mask design. Participants in simulations 1–3 also were uncertain about using unfamiliar designs even though they had the opportunity to decipher cues by comparing the current design with the design they just donned and doffed.

In addition to inhibiting proper doffing, unfamiliar PPE designs sometimes required HCP to change doffing order and doffing strategies. To correctly doff the simulation 4 gown, for example, HCP must first don the gown over their heads, place their thumbs through the thumb loops, and don gloves over the loops. HCP should doff by breaking the gown’s neck closure first, then remove the gown and gloves together. Participants felt this process required a different mental approach than that required to remove the gown and gloves separately.

Participants identified several factors that influenced their general doffing strategies, most prominently the desire to avoid self-contamination and patient care demands related to their specific roles. They acknowledged these concerns when doffing any PPE item but noted additional cognitive demands when using unfamiliar PPE.

### Theme 2. Influence of routine practice

Participants who encountered unfamiliar PPE during simulations described their approaches to troubleshooting the appropriate doffing sequence and strategy. They particularly noted the role of their day-to-day PPE practices including connected factors such as designs typically worn (and donning/doffing order) and design cues (eg, fasteners or perforations). Participants also cited less obvious factors such as donning and doffing frequency during routine patient care tasks and the specific contexts in which they provided care. Many stated that their routine practices, which they developed through frequent donning and doffing, ingrained habits into their muscle memory. When encountering unfamiliar PPE, they relied on these habits rather than on conscious thought. Some perceived these habits as potentially difficult to change.

The 20 participants (10 per hospital) in simulation 4 with SIU training often drew on this training when formulating strategies for doffing unfamiliar PPE. The specialized training included practice donning and doffing unfamiliar PPE items and more complex ensembles, practice following more rigorous donning and doffing protocols, and coaching with feedback. Several participants described how the rigorous protocols they followed as part of SIU training (and/or subsequent biocontainment unit experience) made them more cautious when doffing and shaped how they conceptualized their risk for self-contamination. They perceived themselves as acting with similar caution in their approach to unfamiliar PPE during simulations. However, SIU-trained participants also acknowledged aspects of donning and doffing in SIU training and the biocontainment unit that differed significantly from the routine practice settings in which they might encounter unfamiliar PPE. These included following steps in a directed donning and doffing process while being coached by an observer, focusing exclusively on donning and doffing, and using specific PPE items and designs. In contrast, participants typically described donning and doffing without coaching while managing competing cognitive demands (eg, conversations, interruptions) during routine practice.

Despite these differences, participants described specific doffing strategies that they used in the simulation as related to their SIU training such as rolling dirty gown surfaces away from their bodies, breaking (rather than untying) mask ties, and using glove-in-glove technique. This was also true when the PPE provided differed from that used in SIU training or the biocontainment unit. For example, one SIU-trained participant intentionally removed the surgical mask ties during the simulation based on the order (s)he was taught to follow for an N95.

Participants in all simulations reported looking for design cues on unfamiliar PPE. Although we focused primarily on doffing, we observed that participants often surveyed PPE while donning to identify cues such as gown tie placement and neck closure, mask fasteners, and Doffy glove tabs. Participants reported looking for design features that resembled or served the same function as those on familiar PPE to inform their doffing strategy. Nevertheless, recognizable cues did not always point participants to a clear and appropriate strategy. In fact, some participants experimented while donning and/or changed their processes between doffing episodes within the same simulation. The cues provided a starting place. Conversely, participants became confused if they did not identify cues or encountered unfamiliar cues.

### Theme 3. Training experiences and needs

Participants described their routine practices as developing over time, shaped by hospital policies, available PPE, protocols, the demands of patient care tasks (eg, frequency of donning and doffing), and importantly, previous training. Many referenced PPE training, including training on multiple designs, that they received during their education, professional training, or employment orientation at a specific hospital. However, given the time elapsed since training, some participants had difficulty recalling the strategies they learned or indeed if training was the source of specific habits. Others described learning different ways to properly doff an item, developing a preferred method, and then using that method with subsequent designs. Though previous training was a touchpoint for participants’ routine practices, a few also noted that Centers for Disease Control and Prevention (CDC) guidelines were available for reference when they were uncertain about donning and doffing protocols.

Participants also recalled that their training provided general guidelines about donning and doffing order, regardless of PPE designs; however, the order participants were taught differed. For example, during simulation 2 (ie, 2 gown designs), a nurse (hospital A) shared, “… always starting with the gown is what we’ve been told.” Conversely, a student (hospital A) assigned to the same simulation stated she was instructed to don gloves first, then the gown. Participants’ comments and strategies indicated that PPE training is not standardized. In addition, some reported that they did not receive either ongoing PPE training that included diverse designs, nor just-in-time training when unfamiliar PPE was introduced. Participants perceived lack of training as a barrier to their ability to doff unfamiliar PPE properly and minimize self-contamination.

Participants felt that training could increase their familiarity with different PPE designs and decrease their likelihood of self-contamination. For example, while watching his performance, a student (hospital A) said, “… just in general, without really kind of any training, doing that I would just assume that I would contaminate myself.” Participants referenced annual competencies required for nursing staff, videos, and posters displayed on units as existing training methods in their hospitals, and they provided feedback to improve training effectiveness. They particularly perceived training that involved physical practice donning and doffing various PPE designs as valuable, compared with either written or verbal instructions or observations of another HCP’s performance.

## Discussion

We qualitatively explored how HCP approach unfamiliar PPE within the context of a mixed-methods study examining factors that influence HCP doffing strategies and self-contamination. We did not include this question in the initial study aims; however, it was implicit in that we included multiple designs of each PPE item in simulations 1–3. We recognized the need to explicitly address it in our analyses after it emerged inductively in participants’ comments during simulations and interviews. Our findings indicate that HCP drew on their routine practices with familiar PPE to inform their strategies. Furthermore, our data suggest that these routine practices develop through prior training and in response to hospital policies and patient care contexts. Thus, HCP feedback on training modalities could improve training and, thereby, likely improve HCP doffing strategies and decrease the risk of self-contamination.

In our previous work, HCP tried to balance doffing PPE safely to reduce self-contamination with patient care needs and the demanding clinical environments in which they work.^9^ However, we found that HCP had clear design preferences, suggesting that HCP do not view different designs of the same PPE item as interchangeable.^16^ HCP who replicate, or adapt, their routine practices and training to unfamiliar PPE may use inappropriate doffing processes and contaminate themselves, particularly if they wear more PPE items during a given patient care episode than usual.^17^

Previous research has likewise demonstrated that different PPE designs are associated with different rates of self-contamination.^18–20^ Hospitals that switch out a specific design for another or do not provide training when new PPE is introduced place the onus on busy HCP to do their own research or to trouble-shoot in the moment. However, the research on training HCP to doff PPE is limited and existing guidelines and recommendations vary.^8,21,22^ CDC guidelines present general donning and doffing instructions but may not include specific designs such as gowns with different neck closures or nondisposable gowns.^23^ In part, this is likely due to the commercial availability of multiple designs, whereas CDC guidelines must cut across all designs. Just-in-time training may help HCP learn to use new PPE designs if buying contracts change or shortages occur, but our findings indicate that HCP could benefit from refresher training sessions, and from training involving actual donning and doffing, rather than written or visual components alone.

Finally, HCP reported that encounters with unfamiliar designs were not unusual, even before 2020. The COVID-19 pandemic exacerbated this problem, given severe PPE shortages and that HCP needed to wear items that were not part of their prior routine PPE ensembles (eg, eye protection, respirators), as well as the introduction of extended PPE use. Given the likelihood of future pandemics caused by respiratory pathogens, HCP strategies for doffing unfamiliar PPE (particularly when used in ensembles involving multiple PPE items) have important implications for infection prevention.

This study had several limitations. We observed HCP in simulated rather than real-world settings. Participants knew they were being observed, which may have affected their behavior (ie, Hawthorne effect). Simulations did not incorporate the patient care tasks that HCP routinely perform while doffing. Given the role of muscle memory, real-world observations might illuminate how these tasks affect practices in ways HCP are and are not aware of. As with any self-reported data, participants’ descriptions of their thought processes and routine behaviors were subject to misrepresentation or recall bias. However, the strength of this approach lies in the valuable insights that participants provide into the observed behaviors. We mitigated potential recall bias by conducting interviews immediately following simulations. Finally, we recruited from only 2 hospitals in similar states, and participants within a hospital were exposed to similar PPE designs, protocols, and training. Nevertheless, HCP brought diverse perspectives due to their different disciplines, professional- and PPE-specific training, and employment histories. We did not recruit based on demographic characteristics (eg, age and sex); however, future research should examine whether these factors affect HCP perceptions of PPE and PPE doffing.

Healthcare facilities should consider the challenges inherent in doffing unfamiliar PPE when introducing new designs and not assume that different designs are interchangeable or that optimal donning and doffing methods are intuitive. In cases of shortages or rapid changes in PPE stock, just-in-time training may help HCP adapt to unfamiliar PPE. However, HCP also require ongoing training that emphasizes hands-on practice using appropriate doffing techniques and practice doffing both routinely used and newly introduced PPE designs.
